# The “chicken-leg anastomosis”: Low-cost tissue-realistic simulation model for esophageal atresia training in pediatric surgery

**DOI:** 10.3389/fped.2022.893639

**Published:** 2022-08-30

**Authors:** Francesca Palmisani, Patrick Sezen, Elisabeth Haag, Martin L. Metzelder, Wilfried Krois

**Affiliations:** Department of Pediatric Surgery, Medical University of Vienna, Vienna, Austria

**Keywords:** pediatric surgery, esophageal atresia (EA), training, simulation, anastomosis, surgical skills

## Abstract

**Introduction:**

Shifting the training from the operating room (OR) to simulation models has been proven effective in enhancing patient safety and reducing the learning time to achieve competency and increase the operative efficiency. Currently the field of pediatric surgery only offers few low-cost trainers for specialized training and these feature predominantly artificial and often unrealistic tissue. The aim of this study was to develop an easy access low-cost tissue-realistic simulation model for open training of esophageal atresia and to evaluate the acceptance in trainees and junior pediatric surgeons.

**Materials and methods:**

The model is fashioned using reconfigured chicken skin from a chicken leg. To create a model of esophageal atresia, the chicken skin is dissected off the muscle and reconfigured around a foley catheter balloon to recreate the proximal pouch and a feeding tube to recreate the distal pouch. Surrounding structures such as the tracheo-esophageal fistula and the azygos vein can be easily added, obtaining a realistic esophageal atresia (Type C) prototype. Evaluation of model construction, usage and impact on user were performed by both a self-assessment questionnaire with pre- and post-training questions as well as observer-based variables and a revised Objective Structured Assessment of Technical Skills (OSATS) score.

**Results:**

A total of 10 participants were constructing and using the model at two different timepoints. OSATS score for overall performance was significantly higher (*p* = 0.005, *z* = −2.78) during the second observational period [median (MD): 4,95% confidence interval CI: 3.4, 5.1] compared to the first (MD: 3, 95% CI 2.4, 4.1). Self-reported boost in confidence after model usage for performing future esophageal atresia (EA) repair and bowel anastomosis (BA) in general was significantly higher (EA: *U* = 1, *z* = −2.3, *p* = 0.021, BA: *U* = 1, *z* = −2.41, *p* = 0.016) in participants with more years in training/attending status (EA MD:5, BA MD: 5.5) compared to less experienced participants (EA MD: 1.5, BA: 1).

**Conclusion:**

Our easy access low-cost simulation model represents a feasible and tissue realistic training option to increase surgical performance of pediatric surgical trainees outside the OR.

## Introduction

Training in pediatric surgery notably faces the challenge of acquiring experience in rare diseases, complex procedures and small operating fields (1). In the last decade changes to the health system have disrupted the traditional paradigm of surgical training, where the resident gradually acquires autonomy in the operating room, further hampering the quality of pediatric surgical training (2). Indeed, the increased interest in patient safety and operating room (OR) efficiency resulted in greater involvement of attending surgeons in the OR, reducing the resident’s autonomy (3), whilst duty hours restrictions have concretely hindered the opportunities of involvement in acute/emergency case management. The result is that an increasing number of graduating residents are not sufficiently prepared for independent practice (3).

Simulation training has been proven effective in enhancing surgical skills, reducing the learning time to achieve competency and overall improving patient care (4). As such, interest in this field is currently rising in pediatric surgery training programs (1, 5–7). Ultra-realistic high-end surgical training units are evolving, but are often cost intensive and limited to wealthy clinical institutions. Animal trainings are similarly expensive, other than hard to obtain and ethically questionable (8). Only a few low-cost trainers for specialized training in pediatric surgery are available with predominant artificial and mostly unrealistic tissue (5, 9, 10). Based on these premises, we decided to develop an easy access low-cost tissue-realistic simulation model for esophageal atresia on the basis of a chicken-leg pyeloplasty model published in 2006 (11) and evaluate its effect in enhancing the performance of trainees and junior attending pediatric surgeons.

## Materials and methods

**FIGURE 1:**
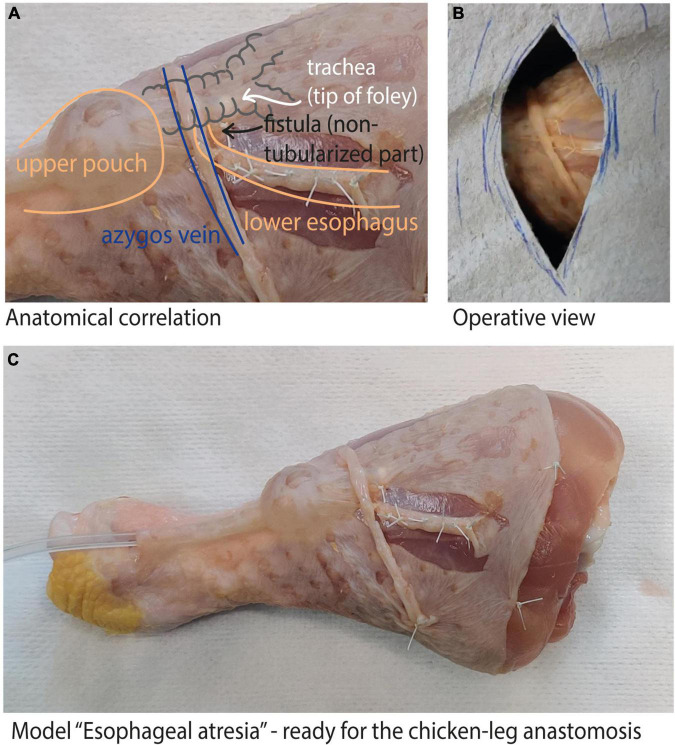
Chicken-leg model: **(A)** shows the anatomical correlation of our model with a type C esophageal atresia; **(B)** shows the operative view once the model is completed; **(C)** complete view of the reconfigured chicken-leg skin to create the esophageal atresia model.

The models are fashioned using reconfigured chicken skin from a chicken leg (Figure 1). To create a model of esophageal atresia, the chicken skin is dissected off the muscle and reconfigured around a foley catheter balloon to recreate the proximal pouch and tubularized around a feeding tube to recreate the distal pouch and tracheo-esophageal fistula (TEF). Surrounding structures such as the azygos vein can be easily added, obtaining a realistic esophageal atresia prototype. Varying the distance between the two ends, as well as adding the presence of a distal or proximal tracheo-esophageal fistula allows the creation of any esophageal atresia type. Finally, the use of a disposable kidney-dish further increases operative details by simulating the thoracic cavity and the actual surgical conditions.

The complete list of materials and approximated costs required for the model is described in Table 1.

**TABLE 1:** Shopping list.

Amount	Material	Approx. Cost (€)
*1*	Bio-chicken thigh	1,95
*1*	***Basic surgical instruments set (1x scissors, 2x forceps, 1x needle-holder, 1xscalpel, 2x mosquito forceps)***	*25*
*1*	***Foley catheter (8 or 10Fr)***	*2*
*1*	***Nasogastric feeding tube (6Fr)***	*3*
*1*	disposable kidney dish	0,05
*1*	4/0 Vicryl suture (or similar)	0,5
*1*	6/0 PDS suture (or similar)	8
*1*	sterile gloves	0,5
**Total**		41

The materials marked in bold may be reused multiple times, thus reducing the overall cost of the single training session. The cost of such materials amounts to 73% of the total.

Our study was conducted on a prototype of a type C esophageal atresia (Figure 1A). All residents and junior attending pediatric surgeons of our department were included in the study, as well as the medical students rotating in the pediatric surgery ward at the time of the study. All participants performed the training twice, at a two weeks interval (t1; t2).

The first session began with the view of an instruction video followed by brief comments from the examiners regarding tips and tricks of model construction and esophageal atresia repair. In the second session the participants could view the video once again, but no additional comment was given. The final instruction video was supplemented with the most given comments on creating the model and can be viewed at http://www.pedsurgtraining.com/videos (video “chicken-leg esophageal atresia - assembly instructions”). The complete instruction guide is shown in the Supplementary material 1.

Before each training, participants were asked to give information regarding their surgical experience in bowel anastomosis and esophageal atresia repair and in particular to subjectively evaluate their confidence in performing an esophageal atresia repair on their own. After each session, the candidates were again asked to fill out a questionnaire evaluating the model, as well as their acquired confidence in performing a bowel anastomosis or an esophageal atresia repair (Supplementary material 2). These self-reported items have been then statistically analyzed.

All candidates fashioned their own model and then performed the atresia repair as shown in the training instructions video “chicken-leg esophageal atresia - training instructions” (see URL above).

Two independent examiners, one attending surgeon and one fellow trainee, evaluated their surgical skills according to a revised Objective Structured Assessment of Technical Skills (OSATS) (12, 13), based on tissue and instrument handling, knowledge of procedure and procedural flow. On the model of previous simulation-training studies (14, 15), an “overall performance” ranking was added which should reflect the overall impression and proficiency in the procedure. Furthermore, specific competences related to esophageal atresia repair such as vena azygos ligation, TEF ligation and anastomosis were closely examined. A detailed description of evaluation criteria can be found in Supplementary material 3. The examiners both assessed all datapoints, were blinded to each other’s evaluation and to their previous assessments, but not to the phase of the trial itself. After completed assessment of both raters, scores were combined and mean was calculated by a third party. No rater training took place before the study.

Progress between the two sessions was evaluated.

For statistical calculations we used the free statistical software environment R (R Core Team, version 4.1.2 Bird Hippie; The R Project, Vienna, Austria) (16). Plots were drawn using the package ggplot2 (version 3.3.5) (17). Descriptive statistics included mean and confidence interval (CI) for model construction time as well as anastomosis time and size of incision. Evaluation of differences between timepoints t1 and t2 regarding these variables was done using a paired Wilcoxon signed-rank test. Overall performance has been identified as primary endpoint. The self-reported items from the participant questionnaire and the foreign observed items from the objective evaluation sheet were described by median as well as interquartile range. Differences between timepoints have been evaluated using the paired Wilcoxon signed-rank test as well. For subjective confidence evaluation pre- and post-training we performed a subgroup analysis and formed two groups based on experience status with a chosen threshold that permitted two equal groups of 5 participants. This resulted in a cutoff at the halfway point of training. Due to the lacking OR experience in esophageal atresia repair we decided to name the two groups “novice” and “intermediate,” respectively. For subgroup analysis, testing between groups was carried out using the Wilcoxon rank sum test. The two-sided significance level was P < 0.05.

## Results

**FIGURE 2:**
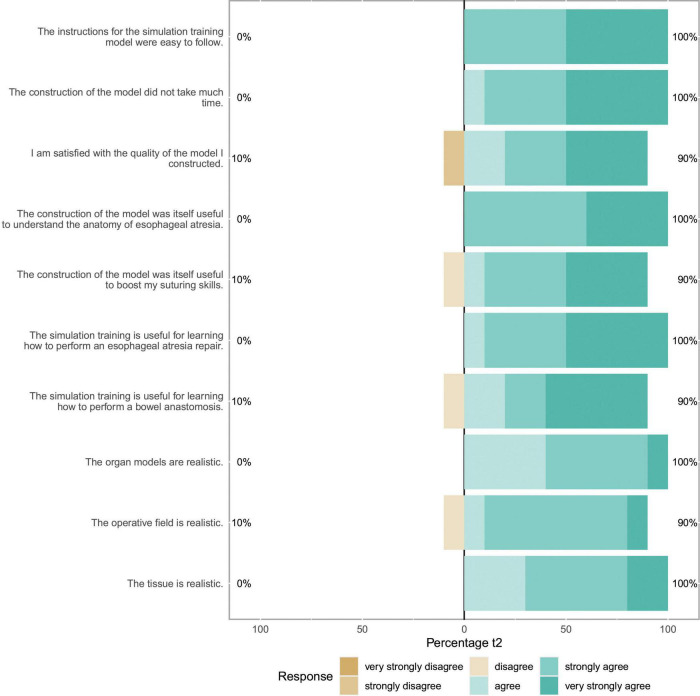
Self assessment items: This Likert plot for t2 shows ten self-assessment items concerning the themes of model assembly, self-assessment of skill acquisition and model realism after completion of the whole training. The scales show the respective fractions of participant answers to the questionnaire. Percentages on the left side show total amount of disagreement and percentages on the right side total amount of agreement with the respective item.

**FIGURE 3:**
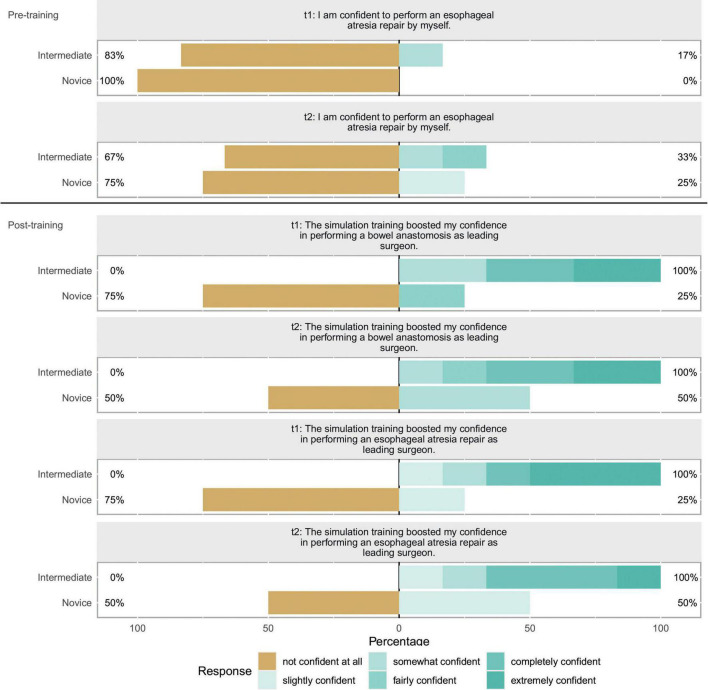
Self-reported confidence evaluation: This Likert shows self-reported confidence items for t1 and t2. The scales show the respective fractions of participant answers to the questionnaire. Percentages on the left side show total amount of disagreement and percentages on the right side total amount of agreement with the respective item. In discordance to the other ten items, these items were scored differently with only one item having a distinctly negative connotation. The respective questions are featured above the bars. Bar color represents the given response according to the legend. A subgroup categorization was carried out for this plot. Participants were separated in “Intermediate” and “Novice” depending on their level of training. The cutoff was chosen so both groups were equally large. Q1 was asked before assembly of the model at both timepoints while Q2 and Q3 were both asked after assembly and usage of the model.

We included 10 participants (8 female, 2 male) for statistical analysis, half of the participants had completed half of their training or had attending status. No participant had already performed an esophageal atresia repair as leading surgeon in the OR. 6 of 10 (60%) had, however, performed at least one bowel anastomosis as leading surgeon. The self-reported items showed no significant change between t1 and t2. Figure 2 depicts the evaluation of the candidates after having completed the whole training (t2), results for t1 are similar. Results of the subgroup analysis for confidence items at t1 and t2 can be seen in Figure 3. A significant boost in confidence is indicated by the Wilcoxon rank-sum test for intermediate participants compared to novice participants. At t1 for confidence in bowel anastomosis intermediate participants reported a median score of 5.5, while novice participants reported a median score of 1, *U* = 1, *z* = -2.41, *p* = 0.016. A similar result can be reported for t2 with a median of 5 for intermediate and a median of 1 for novice participants which resulted in *U* = 2, *z* = -2.07, *p* = 0.038.

For boost in confidence in esophageal atresia repair as a leading surgeon we can report a median score of 5 for intermediate participants and 1.5 for novice participants and the Wilcoxon rank-sum test showing a significant result of *U* = 1, *z* = -2.3, *p* = 0.021. And at t2 median for intermediates was 5 and for novices 2 resulting in *U* = 1, *z* = -2.29, *p* = 0.022.

**FIGURE 4:**
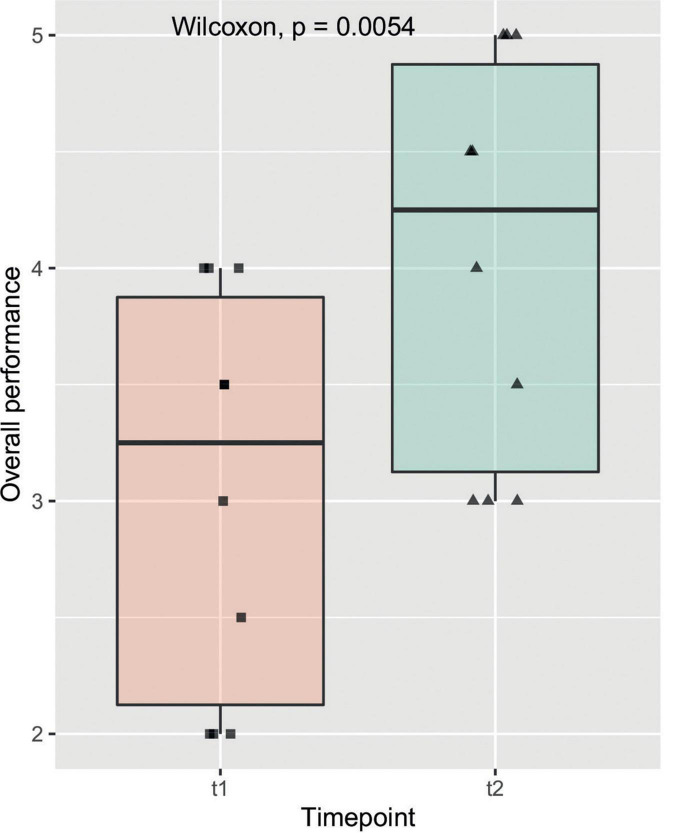
Overall performance: The box plot shows the observed item overall performance on the y axis compared to the two timepoints t1 and t2 on the x axis. The solid line represents the median, the box represents the interquartile range (IQR) and the whiskers represent 1.5x IQR. Individual data points are shown on the plot with a horizontal jitter for better recognition. The result of the Wilcoxon signed rank test is featured here as well as in Table 1.

**FIGURE 5:**
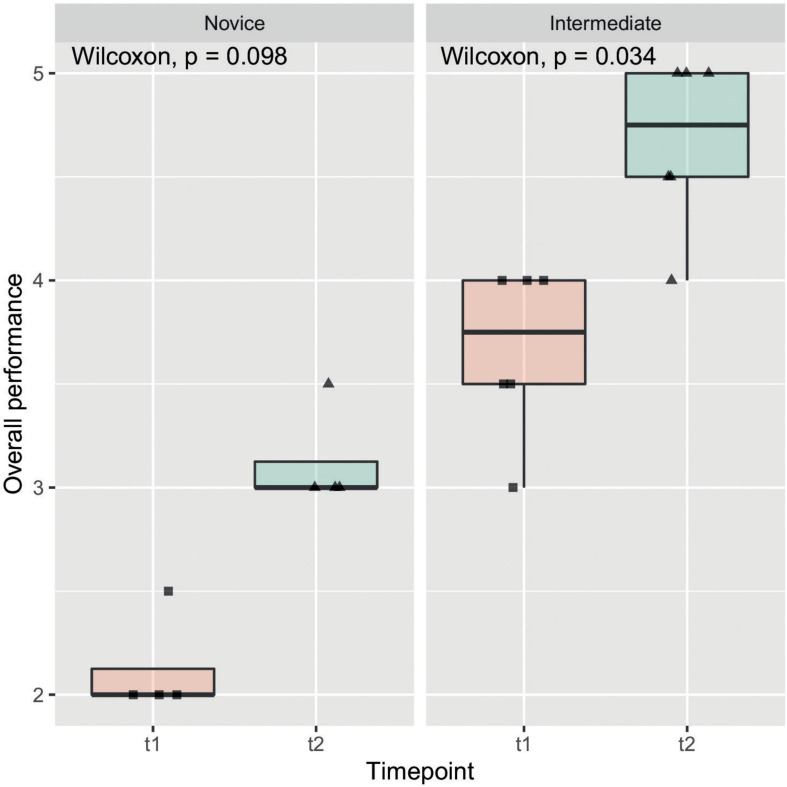
Overall performance subgroup analysis: In this box plot, which shows the same data as in this figure, subgroup analysis by experience of participants was performed. Observed increase in overall performance seemed to have been more pronounced in intermediate participants compared to novice participants.

The objective evaluations regarding time of model-creation, anastomosis, size of incision as well as the OSATS Scores at t1 and t2 are listed in Table 2. All observed items, other than the azygos vein ligation, marked a significant improvement amongst participants from one time-point to another. Figure 4 gives insight on data of the item “Overall performance” in a box plot between timepoints, which shows one of the stronger and more significant improvements over time. This relationship was also examined by means of subgroup analysis as can be seen in Figure 5. Model construction time similarly significantly improved from one visit to another. No significant changes could be observed in the size of the incision and time to anastomosis.

**TABLE 2:** Observer variables.

	t1	t2	*z*	*p*
Model construction time (min)	24 [20,28]	19 [16,22]	2.08	0.038
Anastomosis time (min)	35 [28,41]	28 [23,33]	1.94	0.052
Size of incision (cm × cm)	5 [4,6] × 3 [3,4]	4 [4,5] × 4 [3,5]	−1.19	0.234
Tissue handling	3 [2.2,3.8]	4 [3.4,4.6]	−2.34	0.019
Instrument handling	3 [2.8,3.8]	4 [3.4,4.6]	−2.16	0.031
Knowledge of the procedure	3 [2.1,3.9]	4 [3.6,4.4]	−2.04	0.041
Flow	3 [2.2,4.3]	4 [3.8,4.2]	−2.51	0.012
Specific competence: Azygos vein ligation	3 [2.8,3.2]	4 [2.9,4.6]	−1.76	0.078.
Specific competence: TE fistula ligation	3 [2.4,3.6]	4 [2.9,4.6]	−1.97	0.049
Specific competence: Anastomosis	4 [3.5,4.5]	4 [3.1,4.9]	−1.99	0.046
Overall performance	3 [2.4,4.1]	4 [3.4,5.1]	−2.78	0.005

This table contains collected variables by observers during model construction and usage during the first time-point t1 and the second time-point t2. Metric variables featured in the top part of the table have the mean displayed with 95% confidence interval in angular brackets. For ordinal values in the bottom part of the table the displayed value is the median, angular brackets show the 25^th^ and 75^th^ percentile. For testing between the timepoints, a Wilcoxon signed-rank test was performed, z denotes the Z-Score and p the p-value. Values for the observed variables in the objective evaluation sheet ranged from 1 to 5. With 1 equaling an insufficient performance, 3 featuring a competent display in the respective task and 5 representing a proficient display of skill.

## Discussion

The American Pediatric Surgical Association (APSA) has recently addressed the issue of *dilution of experience* in pediatric surgery, which currently threatens the quality of children’s surgical care (18). Amongst the list of possible solutions, APSA underlined the importance of redefining the paradigm of pediatric surgical training, including in the existing curricula the use of simulators and the organization of simulation-based training (18).

Our initiative derives exactly from the need to reform and adapt the standards of training in our center, to ensure quality of care in the OR, while assuring that graduating residents gain enough experience for future independent practice, with adequate technical skills recuperated outside the operating theater.

We opted to design an easy access low- cost model which could be replicated in any setting, in order to enable its use outside duty hours or mandated didactic sessions as well as in lower-income countries. Simulation models from chicken thighs have been validated in various disciplines like neurosurgery and urology and gained a very high acceptance as, unlike synthetic models, reconfigured chicken skin has the advantage of providing excellent approximation to living tissue (11, 19). To our belief, this offers a notable advantage to the use of plastic materials, which do serve as task trainers but lack in tissue realism. To our knowledge, this is the first chicken-leg model tailored for pediatric surgery training.

Esophageal atresia is a rare congenital malformation, with an estimated prevalence of 1–2 in 5000 live births. Its adequate repair requires high surgical skills, including the ability to carefully handle delicate tissues in a very small operating space. Being able to perform an esophageal atresia repair independently is a requirement of most pediatric surgery training programs (20). However, due to the low-volume caseload of the disease, also reference centers do not allow residents to acquire enough experience in the clinical setting only.

We therefore considered esophageal atresia repair as a perfect candidate for simulation training and tested the acceptance of our model amongst the trainees and junior attending surgeons in our center.

All participants found the model useful for training in bowel anastomosis and esophageal atresia repair. It is definitely clear, that this training model is not nearly a substitute for performing and training real operations under close supervision, but in particular at the end of the second training all senior residents declared that the simulation had increased their confidence in performing bowel anastomosis and esophageal atresia repair as leading surgeons.

Independently from the level of training, creating the model and performing the repair itself was considered helpful to improve not only the technical skills, but also the basic understanding of the anatomy of the malformation and the steps of the procedure. In case of junior residents in particular, we believe that this aspect may lead to an improvement of the performance in the OR, as it allows the trainees to consciously assist the procedure, without requiring constant guidance.

The model itself was generally favorably accepted by the candidates. It is to be noted, however, that the extensive dissection of the lower pouch, often required to perform the anastomosis on the model, was highlighted as possible flaw. The candidates expressed the concern that this may lead to confusion in the OR, where the lower pouch actually needs to be dissected as little as possible to secure blood supply to the esophagus (21).

It is moreover to be underlined that dissection of the esophageal pouches from the surrounding mediastinal structures may be extremely insidious, and injuries to critical structures such as trachea and vagus nerve are not uncommon. In these regards our model, similarly to those already described in literature, cannot substitute the guidance of an expert surgeon during the actual esophageal atresia repair.

As a task-trainer for dissection, however, it was appreciated especially due to the realism of the tissue. Indeed, the haptic component of the chicken skin was unanimously well appraised, consistently with previous studies on similar models (11). We believe that this constitutes a significant advantage to the synthetic models currently available (1, 5, 22), although the realism of a multilayered tissue is still missing.

Beside the positive subjective evaluation of the candidates, we could also observe an objective improvement of their performances during the two training sessions (t1 vs. t2) (Figures 4, 5). Indeed, at t2 all candidates performed the repair in less time and scored higher in the OSATS. A statistically significant improvement was detected in the “intermediate” group, underlining that this training may be particularly helpful for candidates that have already gathered some experience in bowel anastomosis or in assisting an esophageal atresia repair. This correlates well with the traditional assumption that surgical expertise is linked to surgical volume (23). Simulation training offers in this context the advantage of a safe environment and reproducible conditions, which also allows to tailor the frequency of simulation to the needs of the single trainee.

We believe that a central advantage of our model is that the candidates are required to perform the whole procedure with minimal to none guidance, being forced to think like expert surgeons. The acquisition of surgical expertise is indeed not limited by single motor skills, but calls for a broader intellectual practice (24). The always more intensive involvement of attending surgeons in the OR notably hampers the acquisition of independent decision-making, with the risk of retaining the trainees at the level of technicians.

Although promising, our preliminary study has a number of limitations. Validation study of the model by a panel of experts is lacking. Moreover a larger pool of candidates, ideally from different training programs and training levels, would be required to further confirm our preliminary data on the positive effect of the training on surgical skills. External evaluation would also be needed to refine the impartiality of the objective evaluation. Indeed, as underlined by other single-center training studies (25) the fact that the examiners already knew the candidates may have biased their judgment.

The decision to include a peer assessment has been inspired by recent literature (26–28), which indicates that there is good agreement between novice and expert raters and that actually expertise in performing a given procedure is not a prerequisite to assess it. However, as this is still matter of debate (29), it needs to be considered as possible further limitation of the study.

Further studies are required to evaluate the model also for minimal-invasive repair.

## Conclusion

Implementing simulation training programs to the classical curriculum of pediatric surgical training is nowadays necessary to allow graduating residents to meet the required level of surgical skills as a basis for a more effective expert training in the OR. Our easy access low-cost simulation model represents a feasible and tissue realistic option to increase surgical performance outside the OR.

## Data availability statement

The original contributions presented in the study are included in the article/Supplementary material, further inquiries can be directed to the corresponding author.

## Author contributions

WK and FP: study conception and design and acquisition of data. FP, PS, and WK: analysis and interpretation of data and drafting of the manuscript. MM, WK, EH, and PS: critical revision of the manuscript. All authors contributed to the article and approved the submitted version.
