# Supplementary material for: The “chicken-leg anastomosis”: Low-cost tissue-realistic simulation model for esophageal atresia training in pediatric surgery
Source: Front Pediatr. 2022 Aug 30;10:893639. doi: 10.3389/fped.2022.893639 (PMC9468334; doi:10.3389/fped.2022.893639)
Supplement: Supplementary file 1 [file Table_1.docx]

**Participant Questionnaire: Participant N_____**

**1. Age ____**

**2. Gender:** M F

**3. Trainee**  Yes No

If yes: Which year of training?

1 2 3 4 5 6

**4. Attending surgeon** Yes No

If yes: how long have you been an attending pediatric surgeon? ____

**5. Have you performed an esophageal atresia repair as primary surgeon?**

Yes No

If yes, how many?

1-5 5-20 >20

**6. Have you performed any other types of bowel anastomosis as primary surgeon?**

Yes No

If yes, how many? 1-5 5-20 >20

**7. I am confident to perform an esophageal atresia repair by myself.**

1 2 3 4 5 6

(1= not confident at all, 2=slightly confident, 3=somewhat confident, 4=fairly confident, 5=completely confident, 6= extremely confident)

**Construction of the simulation training model**

**1.The instructions for the simulation training model were easy to follow**

**1 2 3 4 5 6**

(1= very strongly disagree, 2= strongly disagree, 3=disagree, 4=agree, 5=strongly agree, 6 = very strongly agree)

**2. The construction of the model did not take much time**

**1 2 3 4 5 6**

(1= very strongly disagree, 2= strongly disagree, 3=disagree, 4=agree, 5=strongly agree, 6 = very strongly agree)

**3. I am satisfied with the quality of the model I constructed**

**1 2 3 4 5 6**

(1= very strongly disagree, 2= strongly disagree, 3=disagree, 4=agree, 5=strongly agree, 6 = very strongly agree)

**4. The construction of the model was itself useful to understand the anatomy of esophageal atresia**

**1 2 3 4 5 6**

(1= very strongly disagree, 2= strongly disagree, 3=disagree, 4=agree, 5=strongly agree, 6 = very strongly agree)

**5. The construction of the model was itself useful to boost my suturing skills**

**1 2 3 4 5 6**

(1= very strongly disagree, 2= strongly disagree, 3=disagree, 4=agree, 5=strongly agree, 6 = very strongly agree)

**After simulation training**

**1. The simulation training is useful for learning how to perform an esophageal atresia repair**

**1 2 3 4 5 6**

(1= very strongly disagree, 2= strongly disagree, 3=disagree, 4=agree, 5=strongly agree, 6 = very strongly agree)

**2. The simulation training is useful for learning how to perform a bowel anastomosis**

**1 2 3 4 5 6**

(1= very strongly disagree, 2= strongly disagree, 3=disagree, 4=agree, 5=strongly agree, 6 = very strongly agree)

**3. The organ models are realistic**

**1 2 3 4 5 6**

(1= very strongly disagree, 2= strongly disagree, 3=disagree, 4=agree, 5=strongly agree, 6 = very strongly agree)

**4. The operative field is realistic**

**1 2 3 4 5 6**

(1= very strongly disagree, 2= strongly disagree, 3=disagree, 4=agree, 5=strongly agree, 6 = very strongly agree)

**5. The tissue is realistic**

**1 2 3 4 5 6**

(1= very strongly disagree, 2= strongly disagree, 3=disagree, 4=agree, 5=strongly agree, 6 = very strongly agree)
