# Supplementary material for: The “chicken-leg anastomosis”: Low-cost tissue-realistic simulation model for esophageal atresia training in pediatric surgery
Source: Front Pediatr. 2022 Aug 30;10:893639. doi: 10.3389/fped.2022.893639 (PMC9468334; doi:10.3389/fped.2022.893639)
Supplement: Supplementary file 2 [file Table_2.docx]

Objective Evaluation Sheet: **Participant No. Model: Start _________ Ende ________ Thoracotomy size:**

**Anastomosis: Start _________ Ende ________**

| **Criteria** | **Level of Competency** | | | | |
| --- | --- | --- | --- | --- | --- |
|  | **Insufficient** |  | **Competent** |  | **Proficient** |
| **Tissue Handling** | 1. Frequent tissue damage due to unnecessary force on tissue or inappropriate use of instruments | 2. No excessive trauma, however no respect of tissue planes, damage to surrounding structures | 3. Careful handling but occasional damage | 4. Careful and efficient handling, rare damage | 5. Consistently handled |
| **Instrument Handling** | Frequently makes tentative or awkward moves with instruments | Elementary use of instruments | Competent use of instruments although occasionally appeared stiff or awkward | Mostly fluid moves | Fluid moves with instruments and no awkwardness |
| **Knowledge of the procedure** | Deficient knowledge. Needed specific instruction at most operative steps | Elementary knowledge, requested guidance often | Knew all important aspects of the operation. | Mostly familiar with the operation | Demonstrated familiarity with all aspects of the operation. |
| **Flow** | Frequently stopped operating or needed to discuss next step | Frequent unnecessary movements, but does not need to discuss next steps | Demonstrated ability for forward planning with steady progression of operative procedure | Mostly fluent and efficient | Obviously planned course of operation with effortless flow from one move to the next. |
| **Specific Competence: Azygos vein ligation** | Insufficient ligation/ dissection, possible bleeding in real life during dissection | Many unnecessary or incorrect movements | Demonstrated ability for correct ligation and dissection | Mostly efficient and careful ligation and dissection | Correct ligation and dissection, efficient and fluent |
| **Specific Competence: TE fistula ligation** | Insufficient ligation, high risk of tracheal leak | Many unnecessary or incorrect movements | Demonstrated ability for correct ligation and dissection | Mostly efficient and careful ligation and dissection | Correct ligation and dissection, efficient and fluent |
| **Specific Competence: Anastomosis** | Insufficient anastomosis | Tissue damaging during anastomosis, incorrect movements | Competent skills in anastomosis sutures | Mostly efficient and symmetric sutures | Efficiently performs a competent anastomosis. |
| **Overall performance** | Unsafe | Safe but incomplete or inefficient | Safe | Competent/safe and efficient | Ready for OR |
