# Supplementary material for: The “chicken-leg anastomosis”: Low-cost tissue-realistic simulation model for esophageal atresia training in pediatric surgery
Source: Front Pediatr. 2022 Aug 30;10:893639. doi: 10.3389/fped.2022.893639 (PMC9468334; doi:10.3389/fped.2022.893639)
Supplement: Supplementary file 3 [file Data_Sheet_1.PDF]

Instruction guide "Esophageal atresia"

http://www.pedsurgtraining.com/

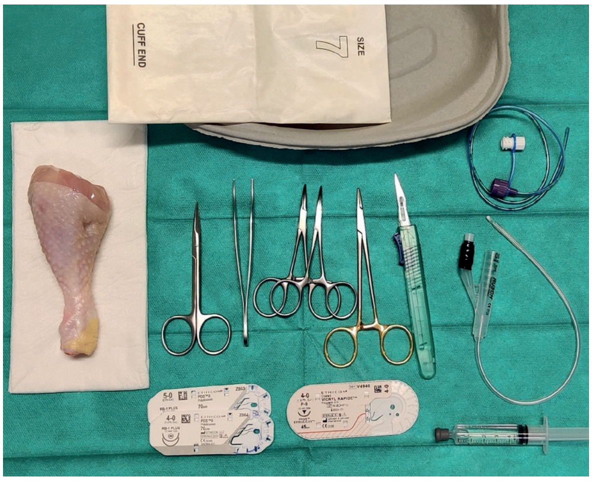

Materials needed (see shopping list)

Shopping list

1x chicken-thigh
1x scissor
1x forceps
1x needle-holder
1x scalpel
2x mosquito forceps
1x inflatable foley-catheter (8 or 10 French)
1x nasogastric tube (6 French)
1x disposable kidney dish
1x 4-0 or 5-0 Vicryl (or similar)
2x 5-0 or 6-0 PDS (or similar)
1x gloves

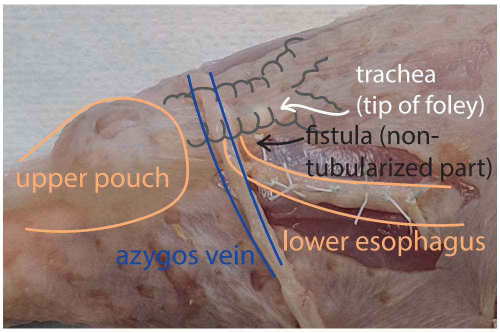

Anatomical correlation

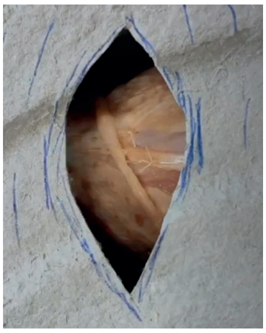

Operative view

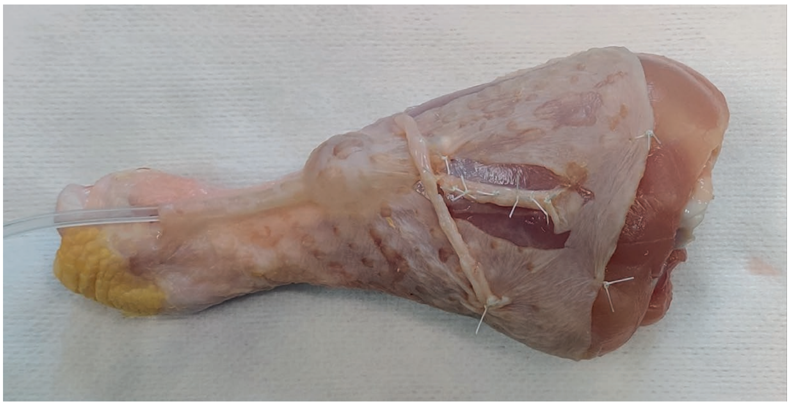

Model "Esophageal atresia" - ready for the chicken-leg anastomosis

Assembly instruction video

Training instructions video

http://www.pedsurgtraining.com/videos

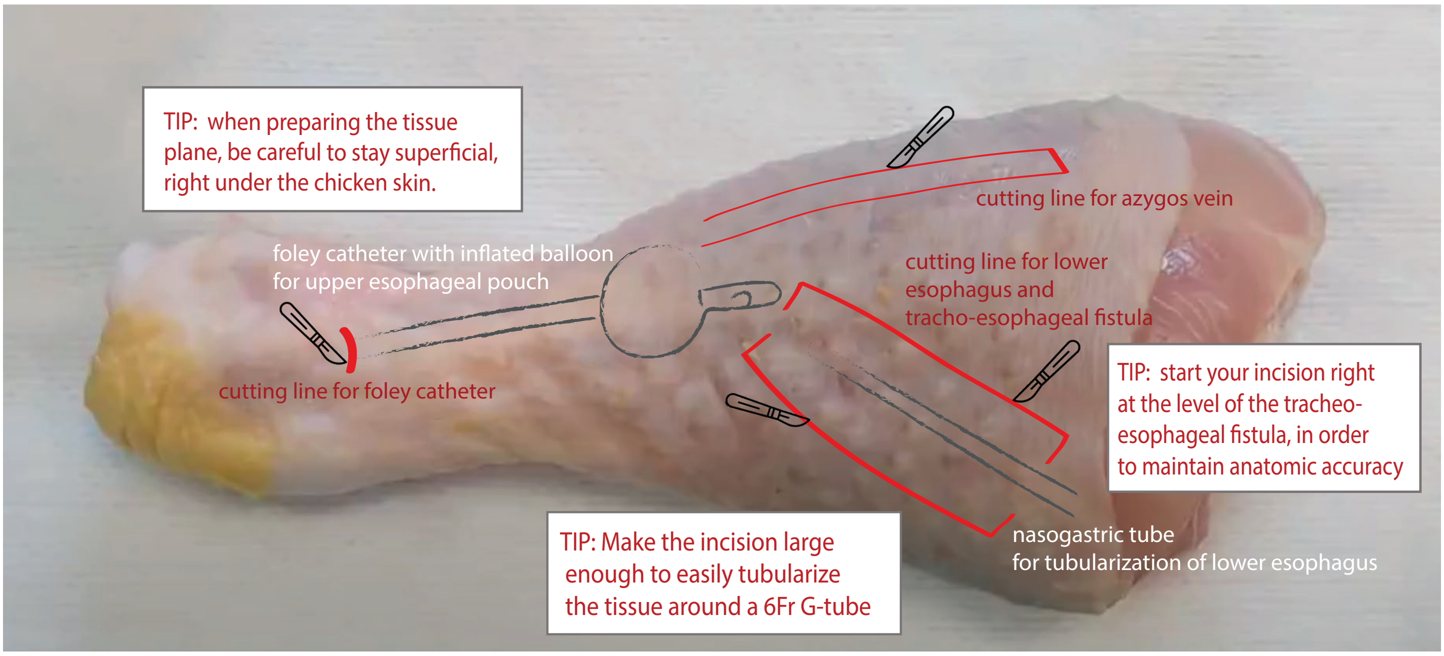

Cutting lines for model creation
